# Chemotypes and Their Stability in *Mentha longifolia* (L.) L.—A Comprehensive Study of Five Accessions

**DOI:** 10.3390/plants10112478

**Published:** 2021-11-16

**Authors:** Katalin Patonay, Helga Szalontai, Péter Radácsi, Éva Zámboriné-Németh

**Affiliations:** 1 Food and Wine Research Institute, Eszterházy Károly Catholic University, Leányka St. 6 Building G, H-3300 Eger, Hungary; szalontai.helga@uni-eszterhazy.hu; 2 Department and Medicinal and Aromatic Plants, Institute of Horticulture, Hungarian University of Agriculture and Life Sciences, Villányi St. 29-43, H-1118 Budapest, Hungary; Radacsi.Peter@uni-mate.hu (P.R.); zamborine.nemeth.eva@uni-mate.hu (É.Z.-N.)

**Keywords:** *Mentha longifolia* (L.) L., horsemint, essential oil, thymol, carvacrol, chemotypes

## Abstract

*Mentha longifolia* (L.) L. is the most widespread wild-growing mint species found, and its chemical composition is extremely diverse. We studied the essential oil (EO) yield, composition, and chemotaxonomy of five, northern Hungarian accessions of the species in a cultivation experiment covering two vegetation years at two parallel sites. The long-term goal is to establish the cultivation of this stress-tolerant species in Hungary as a source of flavoring and preservative agents for commercial use. Essential oil yield (1–2 mL/100 g) was observed to be dependent on both the accession and the year. Accession **HV1** is assumed to be a new, presumably rare chemotype containing carvacrol (19.28–20.56%), 1,8-cineole (14.87–17.45%), thymol (13.36–13.90%), carvacryl acetate (8.81–10.40%), and *para*-cymene (7.24–8.01%). Only minor fluctuations occurred in concentrations of these constituents due to habitats and years. A radical change in essential oil composition was observed in accession **HV2**, as one batch was based on thymol (19.79%) and 1,8-cineole (14.93%), while the others were rich in dihydrocarvone isomers (up to 69%). Although this needs further investigation, it does explain the coexistence of limonene-oxo and *γ*-terpinene pathways in horsemint. According to the literature, the pathway leading to thymol isomers and/or esters may be rare in the entire *Mentha* genus. We also demonstrated that known chemotypes of horsemint may differ in variability of their EO composition. Our results also led to the conclusion that any declaration on chemotype needs detailed examination and is not realistic on the basis of a single sample. Assumptions were made about the potential areas of utilization: beside fragrance and flavoring uses of essential oils free from pulegone and menthofurane, thymol-based ones may be used as antioxidative and anti-spoilage agents.

## 1. Introduction

Horsemint, wild mint or biblical mint (*Mentha longifolia* (L.) L., further: ML) is a perennial herbaceous (hemicryptophyte, H) aromatic plant species belonging to the Mentha section in the *Mentha* genus within the Nepetoideae subfamily of Lamiaceae. The species shows broad genetic variability, including 22 subspecies [1] and many varieties. It is re-ported to be the most widespread wild-growing mint taxon in the world [1,2], as its distribution area covers temperate and subtropical parts of Europe, western and central Asia, and northern and southern Africa. It colonizes a wide variety of habitats, especially in mountainous regions, even up to 3145 m above sea level (*a. s. l*.) [3]. The banks of streams, wet meadows, pastures, and forest glades are suitable for ML as well as ruderal fields [4], temporal streams in the Near East (‘wadi’) or even semiarid areas [3]. ML is also variable in its morphology [1,3,5,6]. Stems are upright, rectangular, green, white-villous, and 40–180 cm tall—depending on abiotic conditions, concurrent plants, and cutting/pasturing. The sessile, white-villous oblong ovate-lanceolate or lanceolate leaves (2–10 cm × 1–3 cm) have coarsely dentate or serrate margins with sharp, irregular teeth [5,6], and bear glandular trichomes containing essential oil (EO). Inflorescences are 2–6 cm spiciform terminal thyrsi with 1–4 mm lilac or (rarely) white corolla and a narrowly campanulate, pubescent calyx having equal or subequal, narrowly triangular or subulate, acute lobes [1,5]. ML is fer-tile. Its fruits are blackish brown, <1 mm diameter nutlets. ML has been known to hybridize with *M. suaveolens*, resulting in the amphiploid mint *M. x spicata* [1]. Thus, ML is one of the species leading to the widely cultivated mint taxon *M. x piperita*, which is again a natural hybrid of *M. aquatica* and *M. x spicata* [7].

The majority of active ingredients of ML belong to two large groups: the shikimates (phenolics, phenoloids) and the mevalonates (terpenes) [8].

**Figure 1:**
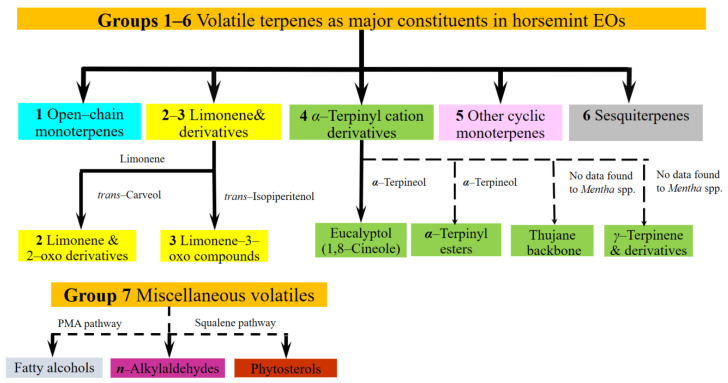
A grouping of *M. longifolia* essential oil constituents based on structure and biogenetic origin, primarily based on Başer et al., 1999 [4], and Llorens-Molina, 2015 [11]. Solid lines indicate frequent components or groups, broken ones symbolize those which are rather rare or detected only once. Individual compound names next to the arrows are important intermediates of the pathway between the initial and the final compound group, based on [11]. ‘No data found for *Mentha* spp.’: the description of the branches of the metabolic pathway of α-terpinyl cation to thujane-backbone monoterpenes and to γ-terpinene derivatives was not found in the literature on *Mentha* spp., although the steps of these pathways are known from other Lamiaceae plants [12,13].

The volatile composition of the species is extraordinarily variable, not only in comparison with the cultivated mints but also amongst wild-growing mint species [9,10]. Data show that the production of these volatiles seems to involve multiple branches of mono-terpene biosynthesis. The composition determined from ML samples of different phenophases and/or organs makes the question more complex [8]. Despite its high natural variability, no comprehensive review of the volatile chemistry of ML has been found. We based our study on the classification of ML EO components established by Başer et al., 1999 [4], adapting and extending their approach to involve other available literature records e.g., [9,10] on the volatile chemistry of the species. The volatiles of ML were organized into seven biogenetic-structural groups (Figure 1). For the purposes of the current study ‘main/major’ means the components over 5% of EO, as determined in some earlier works [2,3].

− **Group 1** Open-chain monoterpenes, usually (if present) linalool [4,9,14,15] and linalyl acetate [16], rarely myrcene [17], and in a single case geranyl acetate [9]. They seldom dominate the EO, and usually accompany derivatives of limonene or α-terpinyl cation.
− **Group 2** Limonene and 2-oxo derivatives: carvone, up to 55–66% [4,14,18,19], and dihydrocarvones, carveoles, and carvyl acetates are typical.
− **Group 3** Limonene-3-oxo derivatives are typical, especially ketones, in general pulegone up to 67–85% [19], menthone, piperitone, piperitenone and 1,2-epoxides, up to ca 55% [3,18]
− **Group 4** Derivatives of α-terpinyl ion *1,8*-cineole [3] accompanies both open-chains and limonene 3-oxo derivatives in concentration 7–44%. Other terpinyl daughter compounds are seldom: α-terpinyl acetate [14,20,21], terpineoles [20], derivatives of γ-terpinene [22,23,24,25,26], and thujane [2,20,21,22,23,24,25,26]. In a single case, borneol [3] was found in high concentration, 29%
− **Group 5** Other cyclic monoterpenes: p-1-menth-2-en-9-ol [26] and rosefuranes [27].
− **Group 6** Sesquiterpenes, usually germacrene D, β-caryophyllene and its oxide [4,11,14]. Concentrations vary on a large scale, 5–29%.
− **Group 7** Miscellaneous: short-chain normal-alkylaldehydes [2]; a C37 fatty alcohol and a phytosterol were reported [28]

In addition to the compound types and their representatives surveyed above, a major unknown was detected [12] in samples originating from the Marmara region of Turkey. The RI, determined on an Innowax FSC column (60 m × 0.25 mm/film thickness 0.25 μm), was 2209. Its main ions, arranged according to decreasing intensity, were m/z 138; 94; 81; 109; 67; 41; 55; 123; 166. (Further GC-MS conditions are provided in [14]). Presence of the unknown was observed in one *M. longifolia* ssp. *longifolia* and six *M. longifolia* ssp. *typhioides* var. *typhioides* EO batches in concentrations of 6–35%. The unknown component was pre-sent in the above batches together with isopulegol, iso-isopulegol, and their acetates (total: 22–85%) which are atypical in ML.

Despite its adaptability, abundance, and various active ingredients, ML is still underutilized in Europe. Consumption of ML is more usual in Turkey, [4,14,24,29] Iran [3], Egypt [16], Tunisia [30], Morocco [31], and KSA [32]. People use it as tea or spice especially for small scale production dairy goods [33] and in folk medicine. Traditional medicinal applications are similar to those of spearmint: digestive complaints, cough, sore throat, and common cold [14,32].

The present work is part of a broader phytochemical study [8,34,35,36] of ML accessions from the northeastern mountainous regions of Hungary. This part of the work aimed to elucidate some characteristics of chemotaxonomy and natural variability of these accessions as potential aromatic crops for the industry. The long-term goal is to establish the cultivation of this stress-tolerant species as a source of low-cost flavoring and as a spoilage inhibitor additive, as well as shelf-life enhancer antioxidants for the food industry. The present study focuses on five ML accessions annotated as **KBÁ**, **HV1**, **HV2**, **EGR3**, and **SZD**. They have been evaluated concerning their EO yield and composition under different ecological conditions over several years to determine their chemotype and the variability of their EO composition during the changing conditions.

## 2. Results and Discussion

### 2.1. Essential Oil Yield Depending on Accession and Year

Table 1 shows the essential oil yield (mL/100 g dry plant material) measured in the five horsemint accessions harvested in the two habitats in 2019 and 2020. Kolmogorov-Smirnov and Shapiro–Wilks tests were significant (*p* < 0.05), that is EO yield data of the subject horsemints does not show Gaussian distribution. The accessions of horsemint were handled as a statistical population sampled twice at different times (2019 and 2020) and under different treatment conditions with respect to their habitats. Thus, samples were handled as related ones in the investigations of significant difference, both when they were arranged on the basis of the year as well as when the habitat was the basis. Therefore, the Wilcoxon signed-rank test was performed on them as a nonparametric analogue of paired *t*-test.

The accession with the highest EO yield is **KBÁ** (1.777–2.077 mL/100 g dry plant material) and the poorest in EO is **EGR3** (0.862–1.253 mL/100 g dry plant material). Previous data on EO yield of the species cover an interval between 0.05 [37]–3.8 mL/100 g [38] but in many cases the plant has been harvested in other or not defined phenological phases. If only the data about plant material collected in flowering stage is regarded, the EO yields determined here correspond to the average 1–2 mL/100 g dry plant material. The result of the Wilcoxon signed-rank tests on the effect of the year is significant, but the direction of difference may be accession dependent. (Z = −2497, *p* = 0.013). In eight cases of the total 10, the 2019 harvest provided higher EO concentration, than that of 2020. The remaining two cases are **KBÁ** plants of SOR (2.055 and 2.077 mL/100 g dry plant material, respectively) and on the other hand **HV1** plants of EGR habitat (1.092 and 1.099 mL/100 g dry plant material, respectively), but in each case the differences are very slight. Among the habitats, there is no significant difference (Z = −0.255, *p* = 0.799), although the two plantations differ in weather conditions, soil type, and age of the plants. Some accessions such as **KBÁ** accumulated significantly more EO at SOR habitat than at EGR. In contrast, other ones, such as accession **SZD**, showed lower EO content in SOR than in the EGR. 

### 2.2. Essential Oil Composition and Its Variability in the Five Accessions

In the GC analyses of the EOs, in total 65 compounds were identified and 14 of them (*cis*- and *trans*-dihydrocarvone, menthone, isomenthone, pulegone, *cis*-piperitone epoxide, *1,8*-cineole, *γ*-terpinene, *para*-cymene, thymol, carvacrol, carvacryl acetate, *β*-caryophyllene, and germacrene D) are major components in at least one sample (Table 2).

**Figure 2:**
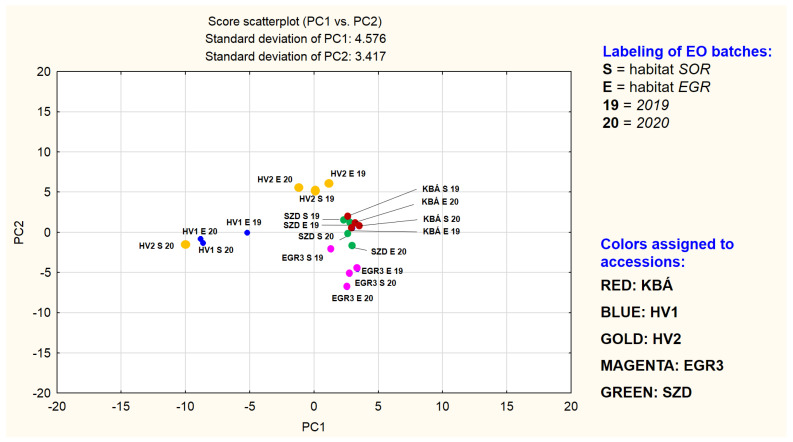
Scatterplot of PC1 vs. PC2.

**Figure 3:**
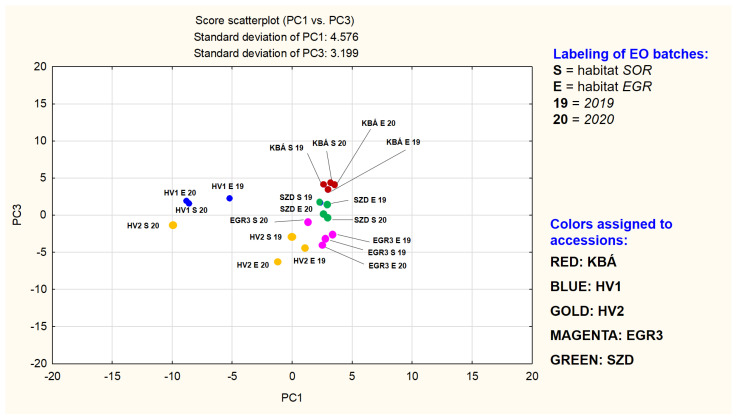
Scatterplot of PC1 vs. PC3.

The total proportion of the identified substances in the EO samples based on area percentages are high, from 96.46 to 99.23%. All EOs were monoterpene rich. Among these, oxygenated monoterpenes are in majority, however, their total percentage is highly variable (53.32–84.86 area% in sum) indicating activity of different branches of the synthesis of monoterpenes in the ML accessions we examined. No open-chain monoterpenes are present as major components. The characteristic cyclic oxygenated monoterpenes in **KBÁ**, **EGR3**, **SZD** and three of the **HV2** samples are typical of the species: limonene-2-oxo, limonene-3-oxo metabolites, and *1,8*-cineole. On the contrary, in the case of all four **HV1** and one of the **HV2** EOs, *γ*-terpinene derivatives are main components. These are highly unusual, not only in the species but also in the entire *Mentha* genus. These samples show the lowest total proportions of oxygenated monoterpenes and highest ratios of monoterpene hydrocarbons as they are enriched in *para*-cymene (the intermediate of thymol isomers/derivatives). The ratio of cyclic sesquiterpene hydrocarbons is relatively high in all the experimental EOs compared to data from the literature. However, the actual concentration is widely variable (11.5–33.41%). Principal component analysis proved to be an appropriate tool for evaluating the variability of the accessions. The first three principal components (PCs) together cover 65.95% of the total variance of the samples. Figure 2 depicts the scatterplot of PC1 vs. PC2. Figure 3 provides the plot of PC1 vs. PC3. Table 3 contains the loadings in PC1-PC3, of those 14 compounds found to be present in concentrations higher than 5% area in at least one sample. Supplement 1 provides the full table of loadings (calculated for the total 65 compounds), plots of them in *loading*(PC1) vs. *loading*(PC2) and *loading*(PC1) vs. *loading*(PC3) planes, component matrix and scree plot of PC1-PC7 completed with percentage of variance explained (Tables S1 and S2, Figures S1–S3).

Figure 2 may demonstrate a grouping primarily based on the mevalonate pathway branches of the main components. The other plot may modify this picture, showing a grouping which depends both on compound type (limonene-2-oxo, limonene-3-oxo, and *γ*-terpinene derivatives) and the accession, except for **HV2**. In the plane of PC1 vs. PC2 points of **HV2** samples rich in dihydrocarvone (HV2_EGR_19, HV2_EGR_20, HV2_SOR_19) form a separate group. The three batches of **HV1** together with the fourth sample of HV2 (HV2_SOR_20) build up another group. Loadings of *γ*-terpinene derivatives in PC1 are all between −0.95…−0.70, in PC2 between −0.11…−0.05. These four EO-s were demonstrated to be rich in 1,8-cineole also. A third range of points, consisting of subgroups less separated from each other (but clearly standing out against the **HV1** and **HV2** point groups) is also detectable and includes **KBÁ, EGR3**, and **SZD**. The EO-s of the three accessions are all based on different subtypes of limonene-3-oxo derivatives. A very tight subgroup is formed by the points representing the four EO batches of accession KBÁ. This indicates that EO composition of **KBÁ** is less influenced by differences of years and habitats. Dominant components of **KBÁ** EO-s are menthone and isomenthone. Next, the less tightly organized subgroup is the set of the limonene-3-oxo-type EO batches consisting of the points of accession EGR3, located in the positive quarter of the plane (PC1 interval cca. 0…+5 and PC2 cca. −7…−2). **EGR3** is dominated by a limonene-3-oxoketone-1,2-epoxide, namely *cis*-piperitone epoxide and two sesquiterpenes. Based on Figure 2 and Figure 3, the evaluation of the uniformity of EO composition in the experimental accessions is also supported. If the presence and concentration of the key components are close to each other in the different batches of the accession investigated, that indicates the essential oil composition is less influenced by yearly and habitat differences. In other words, it is a sign of uniformity of the essential oil composition.

### 2.3. Chemotaxonomic Evaluation of the Studied Accessions

The uniformity of qualitative and quantitative EO composition of accessions investigated here was assessed via the homogeneity of the concentration of the major components in terms of their coefficient of variation (CV). EO samples to a component are evaluated as extremely homogeneous if CV ≤ 10%, homogeneous if 10 < CV ≤ 20%, heterogeneous if 20 < CV [41] and extremely heterogeneous if CV > 50%.

Name, percentage, the CV calculated from the former and homogeneity of the major components in the essential oil batches from each of the accessions are summarized in Table 4.

Accession **KBÁ** produced stable ratios of menthone, isomenthone and *β*-caryophyllene in higher concentrations (49.09–62.65, 5.74–6.74, and 4.72–6.57%, respectively). Thus, these components, being present in proportions less influenced by environmental factors, may be regarded as the ones which determine the chemotype of **KBÁ**. Based on the grouping of Başer and co-authors [4], **KBÁ** can be classified as a limonene-3-oxo ketones/sesquiterpene hydrocarbons-based type characterized by menthone as a dominant constituent accompanied by isomenthone and among the sesquiterpenes, *β*-caryophyllene. The EO profile is completed by pulegone as a major component present in highly variable proportions (5–16%). Table 4 summarizes the major components of **KBÁ** and their homogeneity in terms of CV. In the literature reviewed, no sample of ML with a similar ratio of menthone:isomenthone:pulegone has been found. However, limonene-3-oxo ketones are very typical in the species.

Accession **SZD** can also be classified as a chemotype based on the compound groups of limonene-3-oxo ketones and sesquiterpene hydrocarbons. High CV emerges in menthone, isomenthone, and germacrene D proportions. Thus, **SZD** may be given as a menthone/isomenthone chemotype in which the characteristic volatiles are accompanied by caryophyllene and germacrene D.

**EGR3** EOs were dominated by limonene-3-oxo compounds, epoxides class, basically by cis-piperitone epoxide. Another distinctive trait of **EGR3** in comparison with the other two accessions providing EOs rich in limonene-3-oxo and sesquiterpene compounds is that EGR3 showed the highest (27.93–33.41% of EO) total sesquiterpene content amongst the five accessions. In every harvest of **EGR3** the compounds provided similar proportions of *cis*-piperitone epoxide (44.42–53.47%), *β*-caryophyllene (13.42–16.49%), and germacrene D (8.79–11.10%) is constantly accompanied by a 4–5% of EO concentrations of *1,8*-cineole and a highly variable content of menthone (0.00–5.16%). Based on this data, EGR3 may be characterized as a *cis*-piperitone oxide/*β*-caryophyllene/germacrene D/*1,8*-cineole chemotype. Until now, no previous records on ML EO batch or chemotype based on the *cis* isomer of piperitone epoxide and similarly high proportions of sesquiterpenes have been found by the authors of this study.

As it was observed in the PC plot, three of the **HV2** samples are similar in their composition. They are based on the limonene 2-oxo ketone and sesquiterpene hydrocarbons compound groups. Their main components are cis-dihydrocarvone (47.57–57.06%), trans-dihydrocarvone (9.93–12.28%), β-caryophyllene (5.98–9.95%), and germacrene D (5.28–6.41). Contrary to the three EOs discussed above, sample **HV2**_2020_SOR may be classified as a *γ*-terpinene derivatives/*1,8*-cineole/sesquiterpene hydrocarbon. It is characterized by thymol (19.79%) *1,8*-cineole (14.93%), *β*-caryophyllene (9.92%), and germacrene D (7.29%). In parallel, the proportion of dihydrocarvones declined severely (*cis* isomer to 8.45%, *trans* to 1.43%). This suggests a phenomenon: it is as if **HV2** could turn the mevalonate stream to the direction of the *γ*-terpinene pathway and *1,8*-cineole synthesis instead of the oxidation routes of limonene. Beside the significant changes mentioned, EO samples of **HV2** grown in Eger in 2020 also demonstrated elevated levels concerning the *γ*-terpinene derivatives. The radical turn of the composition emerging in HV2_SOR_2020 made concentration of all major monoterpenes extremely heterogeneous in terms of CV. In terms of the high CVs of the aroma compounds of accession **SZD**, a slight shift in monoterpene biosynthesis may be expected due to some environmental effects. Nevertheless, this phenomenon differs from the fundamental change of EO composition detected in **HV2**. It can also be established that **HV2** demonstrated the ability of the species to maintain both the biosynthetic pathways of *γ*-terpinene and limonene-2-oxo derivatives. This is a new phenomenon, as ML chemotaxa producing *γ*-terpinene derivatives have only been known to exclude this branch of the limonene route until now.

**HV1** EO samples lack both limonene-2-oxo and limonene 3-oxo compounds. Instead of them, carvacrol (19.28–20.56%), *1,8*-cineole (14.87–17.45%), thymol (13.36–13.90%), carvacryl acetate (8.81–10.40%), and *para*-cymene (7.24–8.01%) are its main components, showing narrow concentration intervals. According to our knowledge, **HV1** represents a new chemotype of *Mentha longifolia* (L.) L. based on a previously unknown combination of *1,8*-cineole and untypical, γ-terpinene derived components. This may be assigned as a carvacrol/*1,8*-cineole/thymol/carvacryl acetate/*para*-cymene chemotype. The rank of a separate chemotype is supported by the fact that the EO composition of **HV1** showed only minor fluctuations among habitats, years, and plantations of different ages. CVs calculated from data of **HV1** demonstrated homogeneity or even extreme homogeneity for the main components assigned above.

Although some accessions with the potential of synthesizing *γ*-terpinene reaction products are known from the literature, the new chemotype is clearly distinguishable from them. A Serbian sample [24] consisted of *para*-cymene (14.1%), thymol (13.3%), *1,8*-cineole (6.8%), *γ*-terpinene (5.3%), and β-caryophyllene (5.0%). However, no presence of carvacrol and carvacrol acetate was reported in this batch. Another reference from the Czech Republic [23] deals with much higher concentrations of *1,8*-cineole (25.0%), thymol (18.6%), and *γ*-terpinene (12.1%) than **HV1**. This cineole and thymol-rich EO profile was completed by *para*-cymene (9.2%) but neither carvacrol nor its esters were detected (in contrast with HV1). Leaf EO of a Turkish accession [23] contained both thymol and carvacrol in the same order of magnitude as **HV1**, but it was characterized by the dominance of piperitone epoxide (55.3%, no isomer assigned). A review of the chemistry of Lamiaceae in Iran [26] reported two *M. longifolia* var. *chlorodictya* Rech. f. EO samples containing carvacrol (4–8%) together with high concentrations (37.1 and 62.1%) of *para*-menth-1-en-9-ol, which again is totally different from the composition of **HV1**.

## 3. Conclusions

Our results carried out with five ML accessions on two habitats over two years enabled us to describe their characteristic volatile profile and its variability regarding differences in experimental habitat and year. To our knowledge, this is the first cultivation experiment of the species involving multiple years and cultivation sites. It has been demonstrated that although their original natural habitats are not further than 5–60 km from each other, their EO spectrum reflected specific features. Three of the accessions could be identified according to the known chemotaxonomical system provided in [4,11]. One (**HV2**) exhibited unexpected changes in its essential oil profile in one year and habitat; thus, no chemotype definition could be determined. In the case of **HV1**, the authors detected an EO profile which seems to represent a new chemotype. The pathway leading to thymol isomers and/or esters may be extremely rare not only in the species discussed, but in the entire *Mentha* genus. The reviews of the EO chemistry of *Mentha* spp. as [9,15,42,43] or the recent work summarizing main metabolite pathways of *M. longifolia* [11] do not mention *γ*-terpinene, *para*-cymene, and thymol isomers at all. The exact steps of the *γ*-terpinene pathway in mints have not been demonstrated until now, either. Beyond the sporadic records referred to earlier of ML EOs based on these components, till 30 August 2021 only a single study was found to describe a γ-terpinene derivative (49% carvacrol) as a dominant volatile in an Iranian wild-grown *M*. x *spicata* population [44].

For the food industry, accessions free from pulegone and menthofurane could be profitable because of the EU regulation limits of these compounds [45]. **HV1, HV2, EGR3**, and **SZD** fulfil the requirement as they do not contain the referenced aromas at all. Unfortunately, accession **KBÁ**, which accumulated the highest EO content amongst the five accessions, may be rejected here because of the relatively high and strongly fluctuating (8–15%) concentrations of pulegone in the EO.

Three accessions demonstrated stable proportions of their main EO components, which led us to suppose that they are largely genetically fixed. Accession **SZD**, however, showed a much larger scattering of the data around years and habitats. This suggests a greater dependence of this taxon on environmental conditions, especially weather, or the influence of the age of the plant. The significant change of EO components in accession **HV2** seems to be a new phenomenon. The background of this still needs explanation, as neither obvious marks of disease or stress nor any signs of disturbed development were visible on the individuals. The shift in its mevalonate pathway may demonstrate a strong adaptivity of **HV2** and calls attention to the fact that detection of the influencing factors which might lead to this behavior must be cleared up in order to ensure the desired quality—or even to promote intended changes in the aroma profile in the future.

The stable proportions of components in the essential oil of the target accession are necessary for producing raw plant material of desired quality in cultivation. In the present study, the most stable ones were **HV1** and **EGR3** throughout years and habitats. Both fluctuations in the aroma profile of the other two accessions in the batches we investigated, and the above-mentioned turn of monoterpene biosynthesis in **HV2** showed that any declaration on chemotype must be well established and needs thorough examination. Beside standardizing the phenological phase, cultivation and processing conditions, it is necessary to check the performance of the genotype under a series of different ecological conditions. Chemotype determination is not realistic on the base of a single sample as unfortunately frequently happens, according to the literature (e.g., [24] or [31]).

The detected terpenic components in these essential oils also reflect broad potential for industrial utilizations of ML in Europe. In the case of the epoxide- and sesquiterpene-rich **EGR3**, the dihydrocarvone-rich **HV2** and of **SZD** the plausible utilization may be to process flavoring and/or odorating agents for food, cosmetic, household, or the fine chemical industry. It can cover the native oils themselves as they contain a variety of components bearing different types and hues [42,46,47] of minty scents and tastes. Thus, these may be appropriate in chewing gums, confectioneries, or oral hygiene products, for example. In the latter, antimicrobial properties of limonene derivatives are also advantageous [8]. Dihydrocarvone has been described as fragrance component and as an important molecule in organic fine chemistry for the synthesis of many other monoterpene fragrances [42]. In the EO of **HV1** and in some cases of **HV2** the thymol isomers may provide additional benefits based on their radical scavenging-antioxidant and broad-spectrum antimicrobial and antifungal effects [12,24,48].

## 4. Materials and Methods

### 4.1. Plant Material

**Figure 4:**
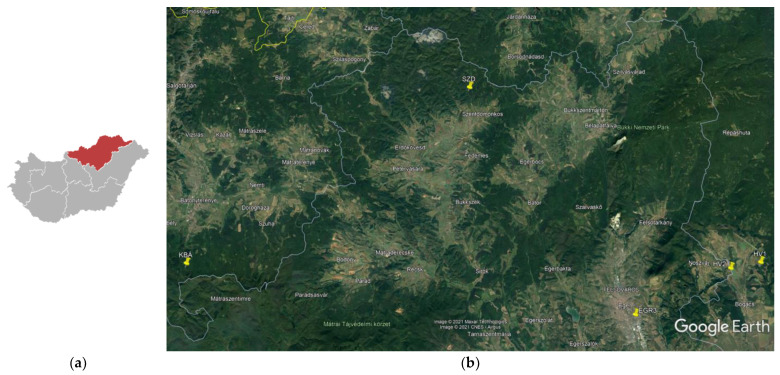
(**a**) A sketch map of Hungary with red highlighting of the Northern region. (**b**) Detailed map of the original locations of the investigated horsemint accessions (yellow line assigns the state border, grey lines are municipal borders). Mapping was performed with Google Earth Pro software package.

Five accessions of ML were selected from thirty-six wild-growing ML populations which had been previously studied, in order to elaborate an optimal method of polar extraction regarding in vitro antioxidant properties [34]. Selected accessions, together with the name, region, GPS coordinates and a short characterization of the collection sites are provided in Supplement (Table S3) and Figure 4a,b.

Identification of the species was performed by H. Szalontai (accessions **HV1**, **HV2**, and **SZD**) and E. Pénzesné Kónya (accessions **EGR3** and **KBÁ**). Voucher specimens, taken in 2019 at full bloom, were deposited in the chemotaxonomical collection (EGR-CH) in the herbarium (EGR) of Eszterházy Károly Catholic University, EKCU.

### 4.2. Experimental Design, Propagation and Maintenance of Plots

Habitat Eger (EGR) plantation were established on 16 May 2018 in the southern part of the botanical garden of the EKCU. (N 47.906834°; E 20.388889°, 226 m *a. s. l*., soil type: heavy loam). The 35–40 cm long shoots with roots were collected in early May 2018 in vegetative phase in the wild populations. Each of the five ML accessions were planted in single rows, to 1 m row and 20 cm plant distance. Right after plantation, the shoots were cut back to a uniform 20 cm to stimulate branching. During the vegetation period the plot was weeded manually, irrigated only in periods of at least a week without any precipitation, and received no additional fertilizer. Acetamipride in a 20 m/m% granulate (‘Mospilan SG 20’, Sumi Agro, Japan) was used against aphids in May. Other pesticide treatment was not necessary in EGR plantation.

The plantation at habitat SOR has been established in late April 2019 at the Experimental Station of Hungarian University of Agricultural and Life Sciences, located in Budapest-Soroksár (N 47.398820, E 19.149270, 100 m a. s. l.; soil type: sandy loam). Rooted shoots of the EGR plantation were used for propagation in vegetative stage. The accessions were planted in 2 × 2 m plots, maintaining 50 cm row and 20 cm plant distance. In the second year, the plant distances disappeared and dense rows were available at both habitats. Maintenance of the plots was carried out as in EGR. Sampling dates of the two years are provided in Supplement (Table S4).

According to a botanical screening published in 2017 [49,50], EGR plot may be characterized by mild climate with a shift to the sub-continental features, with hot summer, not too harsh winter: average annual temperature is 9.9 °C. Usually July and August are the warmest months. Frost-free period in general is mid-May till late September. Meteorological data of habitat EGR from the blooming period of the plants in 2019 and 2020 are provided by the National Meteorological Service of Hungary (Station №.: 53215, ID ‘Eger-Tanárképző Főiskola’, location N 47.903889°; E 20.388889; 225.2 m *a.s.l*.) Proximity of urban heat island may elevate the daily temperature minima at the plot EGR.

Meteorological data of habitat SOR from the blooming period of the plants in 2019 and 2020 are provided by the station established by the Hungarian University of Agronomical and Life Sciences on the plot. The habitat is characterized by the continental climate of the Hungarian Great Plains, thus, it has a broader interval of the daily temperature. A summary of weather in the blooming period (20 June–25 July) in the two years at the experimental habitats are provided in Supplement (Table S5) Soil of habitat EGR is heavy loam with characteristic high water absorptive ability. N, P, K, and humus contents are significantly higher in this plot than in habitat SOR which has a sandy loam soil. Magnesium, manganese, copper, and zinc ion concentrations are also higher in EGR as in SOR plot. Sodium and sulphate contents demonstrate an opposite trend (Table S6).

### 4.3. Sampling and Preparation to Chemical Analyses

Sampling for determination of EO yield and analyses of EO composition were carried out in both habitats in 2019 and 2020, in the full bloom. Sampling dates are summarized in the Supplement (Table S1).

In each plot, 30–45 shoots were chosen randomly and cut ca 5 cm above the ground. Samples were hung at a dry, shady, room-temperature (RT) place for 21 days to drying. Dry leaves and inflorescences (25–35 g per sample) were collected to paper sachets, closed and until distillation of EO were stored at a dark, dry place at RT. Stems were discarded.

### 4.4. Method of EO Extraction (Distillation), Measurement of Essential Oil Yield

EO was extracted with a Clevenger apparatus. EO yields were determined by gravimetry. In the habitat EGR, EO yield determination was made in triplicate both in 2019 and 2020. In the habitat SOR, EO content determination was performed in duplicate. In result the mean of the replicates are provided. Results are given as ml EO per one hundred gram of dry plant material.

### 4.5. Assessment of Qualitative and Quantitative Composition of Essential Oils

Analyses of ML EO composition were carried out via gas chromatography (GC). Apparatus: Agilent GC 6890, mounted with an HP-5MS capillary column (30 m × 0.25 mm, 0.25 μm film thickness) and an Agilent 5975 inert mass selective detector. Carrier gas: helium, constant flow of 1 mL/min. Operation conditions: splitless injection (volume: 0.2 µL of EO dissolved in *n*-hexane to 1% *v*/*v*), injector (Agilent Technologies 7683B) temperature: 230 °C, transfer line temperature: 240, and detector temperature: 250 °C. Temperature programming: initial, 60 °C, heating at a rate of 3 °C/min up to 240 °C. MS conditions: Ionization energy was 70 eV, mass spectra were recorded in full scan mode, to obtain the total ion current (TIC) chromatograms. Identification of components was performed based on their TIC chromatograms using the spectral collection of Adams [39] and the NIST webbook [40]. Beside the referred public databases, GC-MS identification was aided by a library of mass spectra made on the spot, using standards under GC conditions identical with the parameters at the determination of composition of the essential oil samples. Standards of borneol, camphene, caryophyllene, caryophyllene oxide, carvacrol, carvone, *trans*-carveole, 1,8-cineole, citronellol, citronellyl acetate, *para*-cymene, *cis*-dihydrocarveole, β-elemene, eugenol, geranial/citral A, α-humulene, isomenthone, linalool, limonene, menthol, menthone, β-myrcene, 3-octanol, α-pinene, β-pinene, piperitone, pulegone, α-terpinene, *γ*-terpinene, terpinen-4-ol, thujene, thymol, and sabinene, were purchased from Sigma-Aldrich (EU). Linear retention indices (LRI) were determined on the basis of the data obtained from injection of a mixture of C9-C23 n-alkanes in 1 *v*/*v*% n-hexane solution. The column type and length, film thickness, carrier gas type, its flow, and temperature programming of the GC apparatus here were the same as applied for the measurements on the essential oil samples. The calculation of the linear retention indices from data of n-alkanes were calculated using the generalized equation of van den Dool and Kratz (1963) [50]. The proposals of Bicchi et al. on analysis of essential oils [51] were in sight during the process. Quantitative composition of ML EOs were given in GC area%. The GC analysis of **HV1** sample at Budapest-Soroksár from 2019 (HV1_2019_SOR) is missing due to technical issues. In Table 2 and further, 0 area% stands instead of *not detectable*. 0 values were introduced for statistical calculations.

### 4.6. Statistical Evaluation of Essential Oil Composition and Yield

Essential oil yield data were investigated using the Kolmogorov–Smirnov and Shapiro–Wilk tests to decide if their distribution is Gaussian. Normality and Wilcoxon’s tests were performed with IBM SPSS Ver. 20, preceded by preparation of datasets in MS Excel 2013 to the importation to SPSS.

From the viewpoint of CV of the major components in an accession, EO samples to a component are considered as *extremely homogeneous* if CV ≤ 10%, *homogeneous* if 10 < CV ≤ 20%, *heterogeneous* if 20 < CV [47], and extremely heterogeneous if CV > 50%, as it was mentioned earlier. Homogeneity or extreme homogeneity of the concentration determined to a major compound in each accession was evaluated as an indicator to that under present cultivation conditions the concentration is less influenced by habitat and year, thus, an indicator of stable proportion of components in the volatile profile of the investigated accession. Therefore, these components were assigned as to be plausibly characteristic to the chemotype of the accession discussed.

For calculation of simple descriptive statistical indices median, mean, extrema, coefficient of variation (CV) and the preparation of GC data to principal component analysis (PCA), MS Excel 2013 was applied. PCA (using correlation method) had been ran using IBM SPSS Ver. 20 and TIBCO Statistica Trial Version for Windows (https://www.tibco.com/resources/product-download/tibco-statistica-trial-download-for-windows; accession on 22 October 2021).

## Figures and Tables

**Table 1:** Essential oil yield (mL/100 g dry plant material) of the horsemint accessions in full bloom.

Accession	Year	Habitat EGR		Habitat SOR	
		YIELD Mean (N = 3)	S.D. ^1^	YIELD Mean (N = 2)	S.D.
**KBÁ**	***2019***	**1.84**	0.10	**2.06**	0.08
**HV1**		**1.09**	0.05	**1.33**	0.08
**HV2**		**1.99**	0.06	**2.00**	0.09
**EGR3**		**1.25**	0.05	**1.21**	0.08
**SZD**		**1.32**	0.06	**1.17**	0.00
**KBÁ**	***2020***	**1.78**	0.03	**2.08**	0.13
**HV1**		**1.10**	0.17	**0.94**	0.06
**HV2**		**1.75**	0.09	**1.12**	0.05
**EGR3**		**1.08**	0.05	**0.87**	0.00
**SZD**		**1.25**	0.03	**1.04**	0.05

^1^ S.D. = standard deviation.

**Table 2:** Essential oil composition of the five accessions of horsemint in the cultivation experiment, given in area percentage. Abbreviations in this table are as follows: **FA**: fatty alcohol, **LRI**: linear retention indices, **PMA**: polymalonate, polyketide pathway product, **pw**: pathway, and **t_R_**: retention time (minute). Concentrations above 5% area are shown in bold. Numbers denoting ways of identification (**ID**): **1** public MS databases [39,40] **2** LRIs, and **3** MS library of standards made on the spot.

Name	t_R_, min	LRI	ID	Habitat: EGR											Habitat: SOR								
				*Year: 2019*					*Year: 2020*						*Year: 2019*				*Year: 2020*				
				*KBÁ*	*HV1*	*HV2*	*EGR3*	*SZD*	*KBÁ*	*HV1*	*HV2*	*EGR3*	*SZD*	*KBÁ*	*HV1*	*HV2*	*EGR3*	*SZD*	*KBÁ*	*HV1*	*HV2*	*EGR3*	*SZD*
***α*-Thujene**	5.31	928	**1, 2**	0	0	0.05	0	0	0	0	0	0	0	0	No data available No data available	0.1	0	0	0	0	0	0	0
***α*-Pinene**	5.56	938	**1, 2, 3**	0.26	0.16	0.34	0.16	0.20	0.3	0.57	0.46	0.40	0.36	0.23		0.59	0.49	0.29	0.34	0.66	0.76	0.31	0.26
**Camphene**	5.95	952	**1, 2, 3**	0	0.02	0.10	0	0	0	0.05	0.12	0	0.01	0		0.11	0.01	0.02	0	0.04	0.06	0	0
**Sabinene**	6.52	976	**1, 2, 3**	0.30	0.99	0.35	0.30	0.20	0.30	1.3	0.58	0.60	0.30	0.28		0.57	0.61	0.26	0.33	1.32	1.44	0.52	0.27
***β*-Pinene**	6.64	980	**1, 2, 3**	0.47	1.07	0.55	0.40	0.40	0.50	1.36	0.74	0.70	0.50	0.44		0.9	0.86	0.49	0.55	1.49	1.49	0.63	0.43
***β*-Myrcene**	6.99	994	**1, 2, 3**	0.31	1.15	0.40	0.35	0.30	0.30	1.48	0.62	0.60	0.32	0.29		0.55	0.59	0.27	0.32	1.45	1.44	0.52	0.27
***3*-Octanol**	7.14	1000	**1, 2, 3**	0.23	0.09	0.34	0.06	0.10	0.30	0.17	0.29	0.10	0.10	0.20		0.25	0.07	0	0.21	0.11	0.06	0	0.06
***α*-Phellandrene**	7.43	1008	**1, 2**	0	0.10	0	0	0	0	0.15	0.06	0	0	0		0	0	0	0	0.13	0.13	0	0
***α*-Terpinene**	7.79	1017	**1, 2, 3**	0	0.88	0.09	0	0	0	1.21	0.39	0	0	0		0.1	0	0	0	0.85	2.34	0	0
***para*-Cymene**	8.09	1025	**1, 2, 3**	0	**7.24**	0.38	0	0	0.10	**8.01**	1.66	0	0	0		0.62	0	0	0	**7.67**	**9.36**	0	0
**Limonene**	8.19	1028	**1, 2, 3**	0.41	0.5	2.73	0.62	0.40	0.60	0.59	2.66	1.20	0.64	0.48		3.3	1.32	0.35	0.43	0.5	0.91	1.76	0.36
***1,8*-cineole**	8.38	1033	**1, 2, 3**	2.81	**17.50**	3.06	3.70	1.50	2.60	**14.87**	4.38	5.00	1.54	2.68		4.64	**5.16**	1.57	2.60	**17.10**	**14.90**	4.94	2.17
***(Z)*-Ocimene**	8.5	1036	**1, 2**	0.50	0.25	0.38	0.64	0.30	0.40	0.22	0.32	1.00	0.40	0.32		0.25	0.63	0.17	0.46	0.24	0.37	0.87	0.43
***(E)*-Ocimene**	8.85	1046	**1, 2**	0.06	0.03	0.03	0.06	0	0.10	0.04	0.03	0.10	0.04	0.04		0	0.06	0.02	0.05	0.04	0.05	0.06	0.04
***γ*-Terpinene**	9.2	1055	**1, 2, 3**	0	4.15	0.49	0	0	0	4.74	1.75	0.40	0	0		0.44	0	0	0	3.57	**7.22**	0	0
**Terpinene-*4*-acetate**	9.59	1065	**1, 2**	0.01	0.69	0	0.01	0	0	0.83	0.20	0	0	0		0	0	0	0	0.77	0.59	0.03	0
**Linalool**	10.76	1097	**1, 2, 3**	0	0	0	0	0	0	0.19	0.39	0	0	0		0	0	0	0	0.17	0.71	0	0
***3*-Octyl acetate**	11.58	1120	**1, 2**	0	0.3	0	0	0	0	0.24	0.06	0	0	0		0	0	0	0	0.39	0.18	0	0
**Menthone**	12.84	1147	**1, 2, 3**	**53.90**	0.42	0.32	**0.68**	**64.00**	**49.00**	0.90	0	**6.10**	**55.6**	**62.7**		0.08	0.09	**66.3**	**55.78**	0.41	0	0	**46.67**
**Isomenthone**	13.26	1157	**1, 2, 3**	**6.34**	0	0	0.41	**13.00**	**5.70**	0.20	0	1.00	**15.14**	**6.16**		0	0	**11.70**	**6.74**	0.04	0	0	**9.57**
**Borneol**	13.43	1162	**1, 2, 3**	0	0	0.64	0	0.10	0.20	0	0.73	0	0.22	0		0.42	0	0.22	0	0	0	0	0
***cis*-dehydro-*α*-terpineole**	13.49	1164	**1, 2**	0	0.60	0	0	0	0	0.66	0	0.20	0	0		0	0	0.12	0	0.63	0	0	0
**Menthol**	13.68	1168	**1, 2, 3**	4.41	0	0	0	0.20	3.70	0	0	0	0.30	1.83		0	0	0.09	3.35	0	0	0	0.20
***trans*-Isopulegone**	13.81	1171	**1, 2**	3.23	0	0	0	0.50	4.50	0	0	0	0.52	3.90		0	0	0.56	2.88	0	0	0	0.31
**Terpinene-*4*-ol**	13.96	1174	**1, 2, 3**	0	0.32	0	0	0	0	0.41	0.40	0	0	0		0	0	0	0	0.27	1.11	0.21	0
**Isomenthol**	14.13	1178	**1, 2, 3**	0.08	1.70	0	0	0	0.10	0	0	0	0.06	0		0	0	0.06	0.07	0	0	0	0.10
***α*-Terpineol**	14.55	1189	**1, 2, 3**	0.55	0	0.53	0.79	0	0.50	1.74	0.08	0.90	0.39	0.58		0.58	0.83	0.33	0.48	1.64	1.13	0.74	0.41
***cis*-Dihydrocarvone**	14.74	1194	**1, 2**	0.02	0.22	**57.06**	0.18	0	0.40	1.99	**47.57**	0	0.07	0.04		**54.5**	0	0.47	0	0.31	**8.45**	0.05	0.18
***trans*-Dihydrocarvone**	15.05	1201	**1, 2, 3**	0	0.07	**11.66**	0	0	0.10	0.37	**9.93**	0	0	0		**12.3**	0	0.08	0	0.04	1.43	0	0
**Octanol acetate**	15.22	1205	**1, 2**	0	0	0	0	0	0	0	0	0	0	0		0	0	0	0	0	0.08	0	0
***1,6*-Dihydrocarveol**	15.49	1211	**1, 2**	0	0	0.17	0	0	0	0	0.16	0	0	0		0	0	0	0	0	0	0	0
***trans*-Carveol**	15.67	1215	**1, 2, 3**	0	0	0	0	0	0	0	0.05	0	0	0		0	0	0	0	0	0	0	0
**Carvenone**	15.99	1223	**1, 2**	0	0	0	0.53	0	0	0	0	0.50	0	0		0	0	0	0	0	0	0.44	0
***cis*-Dihydrocarveol**	16.03	1224	**1, 2**	0	0	0	0	0	0	0	0.28	0	0	0		0	0	0	0	0	0	0	0
**Citronellol**	16.12	1225	**1, 2, 3**	0	0	0	0	0	0	0.17	0	0	0	0		0	0	0	0	0.12	0.08	0	0
***cis*-3-Hexenyl isovalerate**	16.24	1229	**1, 2**	0	0	0	0	0	0.10	0.20	0.16	0.1	0.15	0		0	0	0	0.12	0.25	0.28	0	0.15
**Pulegone**	16.38	1232	**1, 2, 3**	**8.45**	0.17	0	0	0.10	**15**	0.76	0	0	0.53	**6.3**		0	0	0.04	**6.59**	0	0	0	0.13
**Carvone**	16.71	1241	**1, 2, 3**	0	0	0.33	0	0	0	0	0.36	0	0	0		0.22	0	0	0	0	0	0	0
**Piperitone**	17.06	1248	**1, 2, 3**	0.08	0	0.25	0	0.70	0.10	0.05	0.28	0	0	0		0.23	0	0.46	0.13	0	0.42	0	0
***cis*-Piperitone epoxide**	17.08	1249	**1, 2**	0	0	0	**53.50**	0	0	0.13	1.80	**44.00**	**2.82**	0		0	**55.34**	0	0	0.14	0.09	**52.10**	**12.20**
**Citronellyl formate**	17.77	1265	**1, 2**	0	0	0	0	0	0	0	0	0.10	0	0		0	0	0	0	0	0	0	0
**Geranial; Citral A**	17.83	1268	**1, 2, 3**	0	0	0	0	0	0	0.08	0	0	0	0		0	0	0	0	0	0	0	0
**Neomenthyl acetate**	17.84	1267	**1, 2**	0.24	0	0	0.09	0.10	0.20	0	0	0.10	0.08	0.14		0	0	0.11	0.21	0	0	0.11	0.14
**Dihydroedulan I.**	18.37	1279	**1, 2**	0.44	0.44	0.44	0.63	0.40	0.20	0.31	0.51	0.50	0.42	0.19		0.36	0.30	0.23	0.22	0.36	0.59	0.38	0.47
**Menthyl acetate**	18.71	1281	**1, 2**	1.18	0	0	0	0	0.90	0	0	0	0	0.5		0	0	0	0.59	0	0	0	0
**Thymol**	18.81	1290	**1, 2, 3**	0	**13.9**	1.34	0.60	0	0.30	**13.75**	4.30	1.10	0.09	0		1.39	0.65	0	0.09	**13.4**	**19.8**	0.69	0.33
**Carvacrol**	19.2	1299	**1, 2, 3**	0	**20.6**	0.46	0.44	0.10	0.30	**20.23**	1.44	0.90	0.14	0		0.11	0.41	0	0.10	**19.3**	1.28	0.61	0.18
**Dihydrocarvyl acetate**	20.02	1321	**1, 2**	0	0	0.3	0	0	0	0	0.23	0	0	0		0	0	0	0	0	0	0	0
**Citronellyl acetate**	20.02	1322	**1, 2, 3**	0	0	0	0	0	0	0.07	0	0	0	0		0	0	0	0	0.1	0.05	0	0
**Thymyl acetate**	21.13	1352	**1, 2**	0	0	0	0	0	0.2	0.19	0.06	0	0	0		0	0	0	0	0	0.64	0	0
**Eugenol**	21.36	1358	**1, 2, 3**	0	0	0	0	0	0	0	0	0	0	0		0	0	0	0	0.12	0.18	0	0
**Carvacryl acetate**	21.95	1374	**1, 2**	0	**10.4**	0	0	0	0	**8.14**	0.43	0	0	0		0	0	0	0	8.81	0.46	0	0
***β*-Bourbonene**	22.26	1383	**1, 2**	0.18	0.03	0.12	0.33	0.2	0.1	0.04	0.14	0.30	0.22	0		0.14	0	0.32	0.13	0.04	0.13	0.21	0.24
***β*-Elemene**	22.55	1391	**1, 2, 3**	0	0	0	0	0	0.1	0.04	0.14	0.30	0.11	0		0	0	0	0.1	0.05	0.09	0.18	0.17
***cis*-Jasmone**	23.09	1405	**1, 2**	0	0	0	0	0	0.1	0.1	0.08	0.10	0.03	0		0	0	0	0.08	0.08	0.34	0	0.02
***β*-Caryophyllene**	23.68	1419	**1, 2, 3**	**6.45**	**9.95**	**5.98**	**16.5**	**6**	4.7	**8.27**	**6.83**	**13**	**6.54**	**5.64**		**7.98**	**15.2**	**5.8**	**6.57**	**10.7**	**9.95**	**16.5**	**9.22**
***α*-Humulene**	25.07	1454	**1, 2, 3**	0.62	0.82	0.23	1.70	0.50	0.50	0.73	0.39	1.60	0.63	0.52		0.51	1.60	0.56	0.68	0.95	0.36	1.67	0.93
***β*-Farnesene**	25.27	1459	**1, 2**	0.27	0.07	0	1.14	0.20	0.30	0.09	0.11	0	0.24	0.19		0	1.07	0	0.31	0	0.14	0	0.35
**Germacrene D**	26.18	1481	**1, 2**	4.92	3.31	**5.88**	**10.9**	**6.70**	3.60	3.36	**6.41**	**11.00**	**7.86**	3.87		**5.28**	**8.79**	**6.51**	**5.82**	3.9	**7.29**	**11.1**	**9.32**
**1-Acetoxy-*p*-menth-3-on**	26.2	1482	**1, 2**	0	0	0	0	0	0	0	0	0.80	0	0		0	0	0	0	0	0	0.52	0
**Bicyclogermacrene**	26.81	1497	**1, 2**	0.75	0	1.50	1.79	1.40	0.50	0.14	1.51	1.80	2.03	0.42		0.8	0.97	1	0.93	0.16	0.69	1.44	1.91
***β*-Cadinene**	29.87	1580	**1, 2**	0	0	0	0.62	0	0.10	0.13	0.44	0.80	0.34	0		0	0	0	0.15	0	0.36	0.70	0.37
**Spathulenol**	29.98	1584	**1, 2**	0.09	0	0.18	0.35	0.10	0.20	0	0.16	0.50	0.2	0		0	0.30	0	0.13	0	0	0.48	0.34
**Caryophyllene oxide**	30.2	1590	**1, 2, 3**	0.18	0.06	0	0.49	0	0.60	0.08	0.12	0.60	0.17	0		0.12	0	0	0.23	0.10	0.12	0.68	0.39
**Viridiflorol**	30.49	1598	**1, 2**	1.11	0	0	0	0	1.20	0	0	0	0	0.86		0	0	0	1.38	0	0	0	0
***Monoterpenes***				***84.10***	***73.40***	***82.45***	***64.10***	***83.00***	***87.00***	***78.06***	***82.74***	***66.00***	***80.65***	***87.10***		***82.40***	***67.35***	***84.20***	***82.43***	***73.5***	***77.6***	***64.9***	***75.27***
**Hydrocarbons**				**1.46**	**13.40**	**4.93**	**1.54**	**1.10**	**1.60**	**15.75**	**7.74**	**3.20**	**1.55**	**1.27**		**5.76**	**2.58**	**0.85**	**1.49**	**13.8**	**21.3**	**3.00**	**1.15**
Open-chain				0.87	1.43	0.81	1.05	0.60	0.80	1.75	0.97	1.70	0.76	0.65		0.80	1.28	0.46	0.83	1.73	1.86	1.45	0.74
Limonene & byproducts ^1^				1.44	2.72	3.97	1.48	1.30	1.60	3.81	4.44	2.80	1.81	1.43		5.36	3.28	1.39	1.65	3.97	4.60	3.22	1.32
*α*-Terpinyl pw				0	0.98	0.09	0	0	0	1.35	0.45	0	0	0		0.10	0	0	0	0.98	2.47	0	0
*γ*-Terpinene pw				0	11.4	0.87	0	0	0.1	12.75	3.41	0.40	0	0		1.06	0	0	0	**11.20**	**16.60**	0	0
**Oxygenated**				**82.00**	**67.00**	**76.90**	**61.60**	**81.00**	**85.00**	**66.41**	**74.00**	**61.00**	**78.17**	**85.20**		**75.10**	**62.85**	**82.30**	**80.16**	**64.00**	**52.40**	**60.30**	**73.27**
Open-chain, alcohol				0	0	0	0	0	0	0.35	0.39	0	0	0		0	0	0	0	0.29	0.79	0	0
Open-chain, aldehyde				0	0	0	0	0	0	0.08	0	0	0	0		0	0	0	0	0	0	0	0
Open-chain, ester				0	0	0	0	0	0	0.07	0	0.10	0	0		0	0	0	0	0.10	0.05	0	0
Limonene-2-oxo, alcohol				0	0	0.17	0	0	0	0	0.48	0	0	0		0	0	0	0	0	0	0	0
Limonene-2-oxo, ketone				0.02	0.29	**69.05**	0.71	0	0.50	2.36	**57.86**	0.50	0.07	0.04		**67.00**	0	0.55	0	0.35	**9.88**	0.49	0.18
Limonene-3-oxo, alcohol				4.41	0	0	0	0.20	3.70	0	0	0	0.30	1.83		0	0	0.09	3.35	0	0	0	0.20
Limonene-3-oxo, ketone				**72.00**	0.59	0.57	1.09	79.00	**75.00**	1.91	0.28	7.90	**71.79**	**79**		0.31	0.09	**79**	**72.12**	0.45	0.42	0.52	**56.68**
Limonene-3-oxo, epoxide				0	0	0	**53.50**	0	0	0.13	1.80	**44.00**	2.82	0		0	**55.34**	0	0	0.14	0.09	**52.10**	12.20
Limonene-3-oxo, ester				1.42	0	0	0.09	0.10	1.10	0	0	0.10	0.08	0.64		0	0	0.11	0.80	0	0	0.11	0.14
*α*-Terpinyl pw., alcohol				0.55	0.32	1.17	0.79	0.10	0.70	2.15	1.21	0.90	0.61	0.58		1	0.83	0.55	0.48	1.91	2.24	0.95	0.41
*α*-Terpinyl pw., ether				3.25	**17.50**	3.06	3.70	1.50	2.60	**14.87**	4.38	5.00	1.54	2.68		4.64	**5.16**	1.57	2.60	**17.1**	**14.9**	4.94	2.17
*α*-Terpinyl pw., ester				0.01	0.69	0	0.01	0	0	0.83	0.2	0	0	0		0	0	0	0	0.77	0.59	0.03	***0***
*γ*-Terpinene pw, phenol				0	**34.50**	1.8	1.04	0.10	0.60	**33.99**	**5.74**	2.00	0.22	0		1.50	1.060	0	0.19	**32.6**	**21.10**	1.3.00	0.51
*γ*-Terpinene pw, phenol ester				0	**10.40**	0	0	0	0.20	**8.34**	0.49	0	0	0		0	0	0	0	**8.81**	1.10	0	0
***Sesquiterpenes***				***14.60***	***14.20***	***13.89***	***33.80***	***15.00***	***12.00***	***12.98***	***16.34***	***31.00***	***18.39***	***11.50***		***14.80***	***27.93***	***14.20***	***16.51***	***15.90***	***19.50***	***33.40***	***23.26***
**Hydrocarbons**				*13.20*	*14.20*	*13.71*	*33.00*	*15.00*	*9.90*	*12.80*	*15.97*	*29.00*	*17.99*	*10.60*		*14.70*	*27.63*	*14.20*	*14.69*	*15.80*	*19.00*	*31.70*	*22.51*
**Oxygenated**				*1.38*	*0.06*	*0.18*	*0.84*	*0.10*	*2.00*	*0.08*	*0.28*	*1.20*	*0.37*	*0.86*		*0.12*	*0.30*	*0*	*1.74*	*0.10*	*0.12*	*1.16*	*0.73*
***Volatile shikimates***				***0***	***0***	***0***	***0***	***0***	***0***	***0***	***0***	***0***	***0***	***0***		***0***	***0***	***0***	***0***	***0.12***	***0.18***	***0***	***0***
***Volatile PMAs (FA & esters)***				***0.23***	***0.39***	***0.34***	***0.06***	***0.10***	***0.40***	***0.61***	***0.50***	***0.20***	***0.25***	***0.20***		***0.25***	***0.07***	***0***	***0.33***	***0.75***	***0.52***	***0***	***0.21***

^1^ See [1] on byproducts (eg. pinene isomers) from the reaction of limonene synthase on GPP.

**Table 3:** Loadings of the 14 major constituents of the investigated EO samples in the principal components PC1-PC3.

Compound Type	Compound Name	Loadings for Major Compounds Calculated to PC1-PC3.		
		Loading/PC1	Loading/PC2	Loading/PC3
Limonene-2-oxo	***cis*-Dihydrocarvone**	−0.055332	0.727988	−0.627070
Limonene-2-oxo	***trans*-Dihydrocarvone**	−0.044463	0.727524	−0.617217
Limonene-3-oxo	**Menthone**	0.546769	0.169262	0.647878
Limonene-3-oxo	**Isomenthone**	0.492990	0.108175	0.453511
Limonene-3-oxo	**Pulegone**	0.312915	0.149585	0.637364
Limonene-3-oxo	***cis*-Piperitone epoxide**	0.307598	−0.714431	−0.431420
*α*-Terpinyl derivative	***1,8*-Cineole**	−0.934298	−0.193698	0.077340
*γ*-Terpinene derivative	***γ*-Terpinene**	−0.958503	−0.059386	−0.002698
*γ*-Terpinene derivative	***para*-Cymene**	−0.980025	−0.080735	0.105628
*γ*-Terpinene derivative	**Thymol**	−0.972715	−0.094191	0.034730
*γ*-Terpinene derivative	**Carvacrol**	−0.757757	−0.101103	0.243685
*γ*-Terpinene derivative	**Carvacryl acetate**	−0.738520	−0.082496	0.265629
Sesquiterpene	***β*-Caryophyllene**	−0.061425	−0.762830	−0.432633
Sesquiterpene	**Germacrene D**	0.412524	−0.605841	−0.595122

**Table 4:** Major components, their percentage, and its homogeneity in the investigated essential oils. The arrangement of the table is based on the accessions.

Compound	Concentrations (Area%) in Samples				CV, %	Homogeneity
	EGR_2019	EGR_2020	SOR_2019	SOR_2020		
***Accession: KBÁ***						
**Menthone**	53.91	49.09	62.65	55.78	10.15	homogeneous
**Isomenthone**	6.34	5.74	6.16	6.74	6.67	homogeneous
**Pulegone**	8.45	15.48	6.30	6.59	46.62	heterogeneous
***β*-Caryophyllene**	6.45	4.72	5.64	6.57	14.68	homogeneous
**Germacrene D**	4.92	3.56	3.87	5.82	22.74	heterogeneous
***Accession: SZD***						
**Menthone**	64.00	55.60	66.30	46.67	15.35	homogeneous
**Isomenthone**	13.43	15.14	11.68	9.57	19.17	borderline case
**β-Caryophyllene**	6.00	6.54	5.80	9.22	22.99	heterogeneous
**Germacrene D**	6.69	7.86	6.51	9.32	17.07	homogeneous
**cis-Piperitone epoxide**	0.00	2.82	0.00	12.20	154.02	extrem. heterogeneous
***Accession: EGR3***						
**cis-Piperitone epoxide**	53.47	44.42	55.34	19176	9.34	extrem. homogeneous
**β-Caryophyllene**	16.49	13.42	15.20	16.46	9.38	extrem. homogeneous
**Germacrene D**	10.89	10.67	8.79	11.07	10.20	homogeneous
**1,8-cineole**	3.70	4.97	5.16	4.94	14.25	homogeneous
**Menthone**	0.68	6.13	0.09	0.00	171.14	extrem. heterogeneous
***Accession: HV2***						
**cis-Dihydrocarvone**	57.06	47.57	54.51	8.45	54.08	extrem. heterogeneous
**trans-Dihydrocarvone**	11.66	9.93	12.28	1.43	56.99	extrem. heterogeneous
***β*-Caryophyllene**	5.98	6.83	7.98	9.95	22.35	heterogeneous
**Germacrene D**	5.88	6.41	5.28	7.29	13.71	homogeneous
**Thymol**	1.34	4.30	1.39	19.79	131.75	extrem. heterogeneous
**1,8-cineole**	3.06	4.38	4.64	14.93	81.38	extrem. heterogeneous
***γ*-Terpinene**	0.49	1.75	0.44	7.22	130.14	extrem. heterogeneous
***para*-Cymene**	0.38	1.66	0.62	9.36	142.14	extrem. heterogeneous
***Accession: HV1***						
**Carvacrol**	20.56	20.23	No data available	19.28	3.32	extrem. homogeneous
**1,8-cineole**	17.45	14.87		17.11	8.50	extrem. homogeneous
**Thymol**	13.9	13.75		13.36	2.04	extrem. homogeneous
**Carvacryl acetate**	10.40	8.14		8.81	12.72	homogenous
***para*-Cymene**	7.24	8.01		7.67	5.05	extrem. homogeneous
***β*-Caryophyllene**	9.95	8.27		10.66	12.73	homogenous

## Data Availability

Not applicable.

## Supplementary Materials

The following are available online at https://www.mdpi.com/article/10.3390/plants10112478/s1, Figure S1 Loading plot of loading(PC1) vs. loading(PC2), in the figure denoted as p1 and p2. Compounds assigned to numbers 1–65 are listed in Table S1; Figure S2. Loading plot of loading(PC1) vs. loading(PC3) in the figure denoted as p1 and p3. Compounds assigned to numbers 1–65 are listed in Table S1; Figure S3 Scree plot of principal components PC1–PC7 as the ones having highest eingenvalues. The plot also gives the percentage of the total variance explained by them. (total: 88.74%); Table S1. Component matrix of the investigated EO samples: scores of PC1–PC7; Table S2 Loadings of the 65 compounds involved to the GC analyses of the horsemint EOs, full table for PC1–PC7; Table S3. Original locations of the five investigated ML accessions in Northern Hungary; Table S4. Date of sampling of the horsemint accessions in their phenophase of full blooming; Table S5. Weather characteristics of the two habitats in the period of the blooming (20 June–25 July in each year); Table S6. Properties of soil at the two experimental habitats.

## References

[1] Tucker A.O., Naczi R.F.C., Lawrence B.M. (2007). Mentha: An overview of its classification and relationships. Mint: The Genus Mentha—Medicinal and Aromatic Plants—Industrial Profiles.

[2] Vining K.J., Zhang Q., Tucker A.O., Smith C., Davis T.M. (2005). *Mentha longifolia* (L.) L.: A model species for mint genetic research. HortScience.

[3] Moshrefi Araghi A., Nemati H., Azizi M., Moshtaghi N., Shoora M., Hadian J. (2019). Assessment of phytochemical and agro-morphological variability among different wild accessions of *Mentha longifolia* L. cultivated in field condition. Ind. Crops Prod..

[4] Başer K.H.C., Kürkçüoğlu M., Tarimcilar G., Kaynak G. (1999). Essential oils of *Mentha* species from Northern Turkey. J. Essent. Oil Res..

[5] Petrulaitis L., Gudžinskas Z. (2018). What are we conserving? A case study of *Mentha longifolia* and allied species from Lithuania. Botanica.

[6] Kew Royal Botanical Garden The Plants of the World/*Mentha longifolia* L.. http://www.plantsoftheworldonline.org/taxon/urn:lsid:ipni.org:names:450735-1.

[7] Gobert V., Moja S., Colson M., Taberlet P. (2002). Hybridization in the section *Mentha* (Lamiaceae) inferred from AFLP markers. Am. J. Bot..

[8] Patonay K., Németh-Zámboriné É. (2020). Horsemint as a potential raw material for the food industry: Survey on the chemistry of a less studied mint species. Phytochem. Rev..

[9] Lawrence B.M., Lawrence B.M. (2007). Oil composition of other *Mentha* species and hybrids. Mint: The Genus Mentha—Medicinal and Aromatic Plants—Industrial Profiles.

[10] Németh-Zámboriné É., Başer K.H.C., Buchbauer G. (2016). Natural variability of essential oil components. Handbook of Essential Oils.

[11] Llorens-Molina J.A., Vacas S., Castell V., Verdeguer M. (2020). Seasonal variations of essential oils from five accessions of *Mentha longifolia* (L.) L. with selected chemical profiles. J. Essent. Oil Res..

[12] Kowalczyk A., Przychodna M., Sopata S., Bodalska A., Fecka I. (2020). Thymol and thyme essential oil—New insights into selected therapeutic applications. Molecules.

[13] Németh É.Z., Thi Nguyen H. (2020). Thujone, a widely debated volatile compound: What do we know about it?. Phytochem. Rev..

[14] Başer K.H.C., Kürkçüoğlu M., Demirci B., Özek T., Tarimcilar G. (2012). Essential oils of *Mentha* species from Marmara region of Turkey. J. Essent. Oil. Res..

[15] Mimica-Dukić N., Bozin B. (2008). *Mentha* L. species (*Lamiaceae*) as promising sources of bioactive secondary metabolites. Curr. Pharm. Des..

[16] Al-Okbi Y.S., Fadel H.H.M., Mohamed D.A. (2015). Phytochemical constituents, antioxidant and anticancer activity of *Mentha citrata* and *Mentha longifolia*. Res. J. Pharm. Biol. Chem. Sci..

[17] Venskutonis P.R. (1996). A chemotype of *Mentha longifolia* L. from *Lithuania* rich in piperitenone oxide. J. Essent. Oil Res..

[18] Younis M.H.Y., Beshir S.M. (2011). Carvone-rich essential oils from *Mentha longifolia* (L.) Huds. ssp. *schimperi* Briq. and *Mentha spicata* L. grown in Sudan. J. Essent. Oil Res..

[19] Sharopov F.S., Sulaimanova V.A., Setzer W.N. (2012). Essential oil composition of *Mentha longifolia* from wild populations growing in Tajikistan. J. Med. Act. Plants.

[20] Llorens-Molina J.A., García-Rellán D., Vacas S., Bonet A. (2015). Individual sampling approach to study the chemodiversity of volatile and semivolatile compounds of *Mentha longifolia* L. growing wild in Jiloca basin, Spain. Int. J. Biosci..

[21] Kapp K. (2015). Polyphenolic and Essential Oil Composition of *Mentha* and Their Antimicrobial Effect. Ph.D. Thesis.

[22] Ćavar Zeljković S., Šišková J., Komzáková K., De Diego N., Kaffková K., Tarkowski P. (2021). Phenolic compounds and biological activity of selected *Mentha* species. Plants.

[23] Mimica-Dukić N., Kite G., Gasic O., Stajner D., Pavkov R., Jancic R., Fellows L. (1993). Comparative study of volatile constituents and an timicrobial activity of *Mentha* species. Acta Hortic..

[24] Akşit H., Demirtas I., Telci I., Tarımcılar G. (2013). Chemical diversity in essential oil composition of *Mentha longifolia* (L.) *Hudson* subsp. *typhoides* (Briq.) Harley var. *typhoides* from Turkey. J. Essent. Oil Res..

[25] Hassanzadeh M.K., Emami S.A., Asili J. (2011). Review of the essential oil composition of Iranian *Lamiaceae*. J. Essent. Oil Res..

[26] Golparvar R., Hadipanah A., Gheisari M.M., Salehi S., Khaliliazar R., Ghasemi O. (2017). Comparative analysis of chemical composition of *Mentha longifolia* (L.) Huds. J. Herbal. Drugs.

[27] Karasawa D., Erdenechimeg A., Okamoto Y., Tateba H., Shimizu S. (1995). A study on Mongolian Mints. A new chemotype from *Mentha asiatica* Borriss and constituents of *M. arvensis* L. and *M. piperita* L.. J. Essent. Oil Res..

[28] Ali H.M., Elgat W.A.A.A., El-Hefny M., Salem M.Z.M., Taha A.S., Al Farraj D.A., Elshikh M.S., Hatamleh A.A., Abdel-Salam A. (2021). New approach for using of *Mentha longifolia* L. and *Citrus reticulata* L. essential oils as wood-biofungicides: GC-MS, SEM, and MNDO quantum chemical studies. Materials.

[29] Güllüce M., Sahin F., Sokmen M., Ozer H., Daferera D., Sokmen A., Polissiou M., Adiguzel A., Ozkan H. (2007). Antimicrobial and antioxidant properties of essential oils and methanolic extract from *Mentha longifolia* ssp.. longifolia. Food Chem..

[30] Hajlaoui H., Trabelsi N., Noumi E., Snoussi M., Fallah H., Ksouri R., Bakhrouf A. (2009). Biological activities of the essential oils and methanol extract of two cultivated mint species (*Mentha longifolia* and *Mentha pulegium*) used in the Tunisian folkloric medicine. World J. Microbiol. Biotechnol..

[31] Ghoulami S., Il-Idrissi A., Fkih-Tetouani S. (2001). Phytochemical study of *Mentha longifolia* of Morocco. Fitoterapia.

[32] Murad H.A.S., Abdallah H.M., Ali S.S. (2016). *Mentha longifolia* protects against acetic acid induced colitis in rats. J. Ethnopharm..

[33] Tunçtürk M., Tunçtürk R., Sekeroglu N., Ertus M.M., Ozgokce F. (2011). Lead concentrations of herbs used in Van Herby cheese. Nat. Prod. Commun..

[34] Patonay K., Korózs M., Murányi Z., Pénzesné Kónya E. (2017). Polyphenols in northern Hungarian *Mentha longifolia* (L.) L. treated with ultrasonic extraction for potential oenological uses. Turk. J. Agric. For..

[35] Patonay K., Szalontai H., Csugány J., Szabó-Hudák O., Pénzesné Kónya E., Zámboriné Németh É. (2019). Comparison of extraction methods for the assessment of total polyphenol content and in vitro antioxidant capacity of horsemint (*Mentha longifolia* (L.) L.). Appl. Res. Med. Aromat. Plants.

[36] Patonay K., Szabó-Hudák O., Szalontai H., Jánószky M., Kónya E., Németh É. (2020). Extraction and identification of major polyphenol constituents of Northern Hungarian horsemint (*Mentha longifolia* L. (L.)). Acta Biol. Plant. Agriensis.

[37] De Sousa Barros A., de Morais S.M., Travassos F.P.A., Gusmão Pinto Vieira Í., Aragão Craveiro A., Oliveira dos Santos Fontenelle R., Silva Alencar de Menezes J.E., Walber Ferreira da Silva F., Araújo de Sousa H. (2015). Chemical composition and functional properties of essential oils from *Mentha* species. Ind. Crops Prod..

[38] Fleisher A., Fleisher Z. (1991). The essential oils from *Mentha longifolia* growing in Sinai and Israel. J. Essent. Oil. Res..

[39] Adams R.P. (2017). Identification of Essential Oil Components by Gas Chromatography/Mass Spectrometry.

[40] Stein S., Mirokhin Y., Tchekhovskoi D., Maillard G., Mikaia A., Neta P., Sparkman D., White E., Yang X., Zaikin V. (2011). Agilent Techologies NIST Mass Spectral Library Revision 2005. (The NIST Mass Spectrometry Data Center (2011) Standard Reference Database NIST 2.0). The NIST Mass spectral search program for the library was distributed by the The Standard Reference Data Program of The National Institute of Standards and Technology of the United States.

[41] Gosztola B. (2012). Morphological and Chemical Diversity of Different Chamomile (*Matricaria recutita* L.) Populations of the Great Hungarian Plain. (Alföldi Vadon Termő Orvosi Kamilla (*Matricaria recutita* L.) Populációk Diverzitásának Értékelése Morfológiai és Beltartalmi Szempontból). Ph.D. Thesis.

[42] Lawrence B.M., Lawrence B.M. (2007). The composition of commercially important mints. Mint: The Genus Mentha—Medicinal and Aromatic Plants—Industrial Profiles.

[43] Salehi B., Stojanović-Radić Z., Matejić J., Sharopov F., Antolak H., Kręgiel D., Sen S., Sharifi-Rad M., Acharya K., Sharifi-Rad R. (2018). Plants of Genus *Mentha*: From farm to food factory. Plants.

[44] Zeinali H., Arzani A., Razmjoo K., Rezaee M.B. (2005). Evaluation of oil compositions of Iranian mints (*Mentha* spp.). J. Essent. Oil Res..

[45] (2008). Regulation (EC) No 1334/2008 of the European Parliament and of the Council of 16 December 2008 on flavourings and certain food ingredients with flavouring properties for use in and on foods and amending Council Regulation (EEC) No 1601/91, Regulations (EC) No 2232/96 and (EC) No 110/2008 and Directive 2000/13/EC. Off. J. Eur. Union L.

[46] Verghese J. (1980). Dihydrocarvone. Perfum. Flavorist.

[47] Bertoli A., Leonardi M., Krzyzanowska J., Ołeszek W., Pistelli L. (2011). *Mentha longifolia* in vitro cultures as safe source of flavouring ingredients. Acta Biochim. Pol..

[48] Nieto G. (2020). A review on applications and uses of *Thymus* in the food industry. Plants.

[49] Szűcs P., Táborská J., Baranyi G., Pénzes-Kónya E. (2017). Short-term changes in the bryophyte flora in the botanical garden of Eszterházy Károly University (Eger, NE Hungary). Acta Biol. Plant. Agriensis.

[50] Van den Dool H., Kratz P. (1963). A generalization of the retention index system including linear temperature programmed gas-liquid partition chromatography. J. Chrom. A.

[51] Bicchi C., Chaintreau A., Joulain D. (2018). Technical editorial: Identification of flavour and fragrance constituents. Flavour. Fragr. J..

