# Supplementary material for: Chemotypes and Their Stability in Mentha longifolia (L.) L.—A Comprehensive Study of Five Accessions
Source: Plants (Basel). 2021 Nov 16;10(11):2478. doi: 10.3390/plants10112478 (PMC8622948; doi:10.3390/plants10112478)
Supplement: Supplementary file 1 [file plants-10-02478-s001.zip › plants-1427091-supplementary.pdf]

SUPPLEMENT 1

DETAILED RESULTS OF PCA

The full table of loadings calculated to the total 65 compounds), plots of them in *loading*(PC1) vs. *loading*(PC2) and *loading*(PC1) vs. *loading*(PC3) planes and eigenvalue scree plot of PC1–PC7 completed with percentage of variance explained by these PCs are provided here**.** Figure S1, Figure S2, Figure S3, and Table S1, respectively.

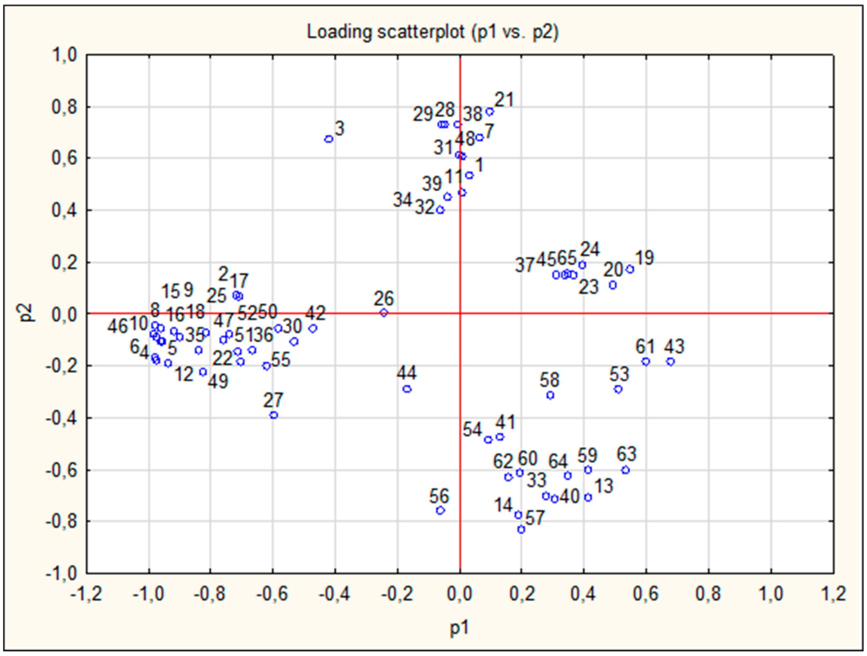

Figure S1 Loading plot of loading(PC1) vs. loading(PC2), in the figure denoted as p1 and p2. Compounds assigned to numbers 1-65 are listed in Table TS1.1.

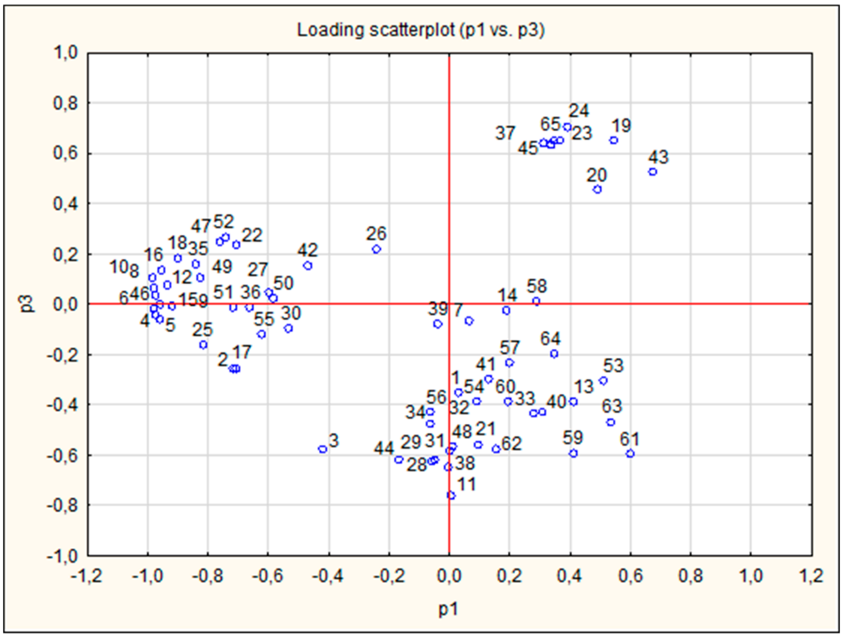

Figure S2. Loading plot of loading(PC1) vs. loading(PC3) in the figure denoted as p1 and p3. Compounds assigned to numbers 1-65 are listed in Table TS1.1

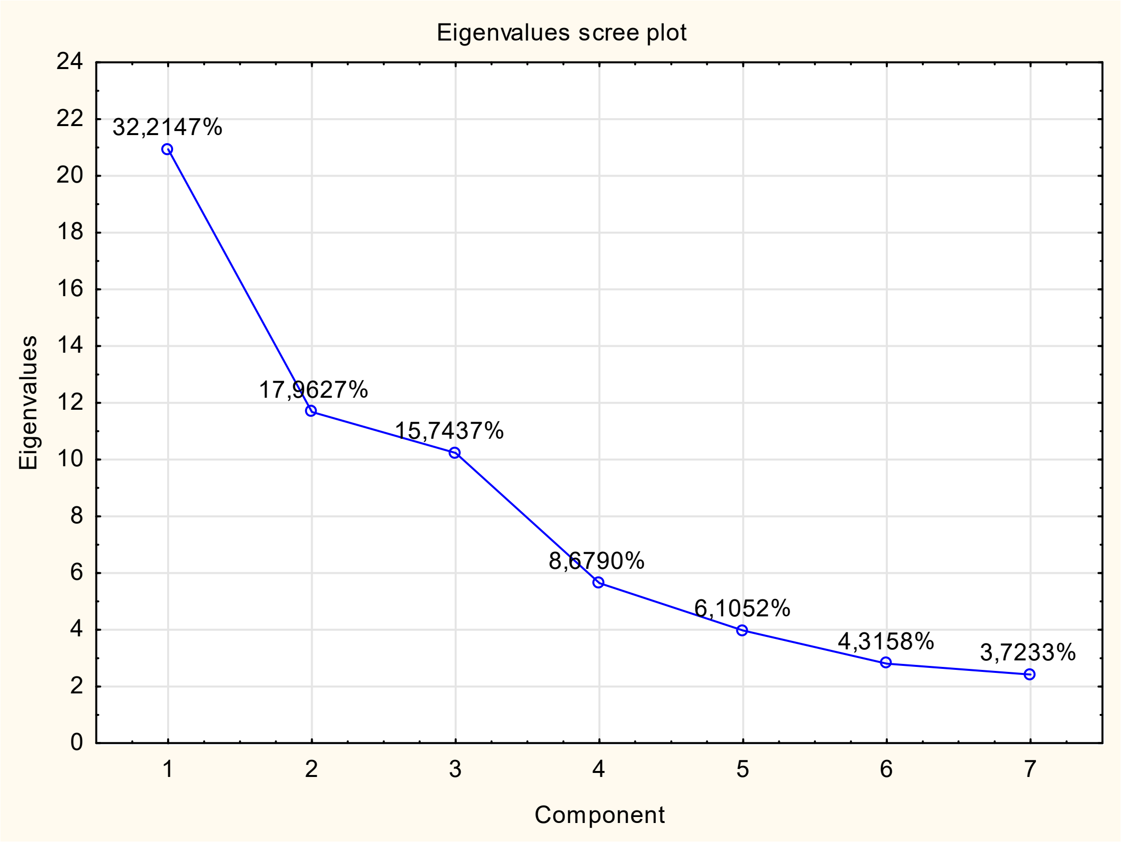

Figure S3 Scree plot of principal components PC1-PC7 as the ones having highest eingenvalues. The plot also gives the percentage of the total variance explained by them. (total: 88.74%)

Table S1. Component matrix of the investigated EO samples: scores of PC1-PC7

**Case label (EO batch name)**	**Principal Component scores**						
	**PC1**	**PC2**	**PC3**	**PC4**	**PC5**	**PC6**	**PC7**
**KBÁ_EGR_19**	3.454	0.873	4.145	-1.555	-1.808	0.353	-0.998
**KBÁ_SOR_19**	2.579	1.843	4.156	-0.152	-0.743	0.682	-0.275
**KBÁ_EGR_20**	3.135	1.124	4.403	-2.773	-2.926	0.218	-0.554
**KBÁ_SOR_20**	2.942	0.538	3.486	-1.794	-1.366	0.027	0.081
**HV1_EGR_19**	-5.227	-0.019	2.282	4.720	0.195	-2.416	-3.563
**HV1_EGR_20**	-8.872	-0.804	2.008	1.807	-1.672	-0.124	2.477
**HV1_SOR_20**	-8.644	-1.274	1.686	1.853	-0.958	0.116	1.529
**HV2_EGR_19**	1.089	6.149	-4.383	1.088	-1.026	1.006	0.375
**HV2_EGR_20**	-1.219	5.569	-6.254	-1.756	-1.637	-3.292	-1.367
**HV2_SOR_19**	0.035	5.115	-2.871	1.735	0.061	3.382	1.298
**HV2_SOR_20**	-10.003	-1.507	-1.291	-6.612	2.638	1.443	-1.357
**EGR3_EGR_19**	3.364	-4.550	-2.503	1.245	0.916	1.162	-2.105
**EGR3_SOR_19**	1.283	-2.079	-0.846	2.192	0.554	3.118	-1.562
**EGR3_EGR_20**	2.535	-6.782	-3.982	-0.885	-2.570	-1.374	1.923
**EGR3_SOR_20**	2.752	-5.186	-3.161	0.685	-0.798	0.360	-0.118
**SZD_EGR_19**	2.884	1.192	1.417	0.111	3.728	-1.111	0.810
**SZD_SOR_19**	2.328	1.655	1.715	0.652	3.649	-0.933	1.451
**SZD_EGR_20**	2.611	-0.173	0.232	-0.346	2.057	-1.331	1.310
**SZD_SOR_20**	2.973	-1.683	-0.238	-0.217	1.705	-1.287	0.645

Table S2 Loadings of the 65 compounds involved to the GC analyses of the horsemint EOs, full table for PC1-PC7.

Name	No.	Loading/PC1	Loading/PC2	Loading/PC3	Loading/PC4	Loading/PC5	Loading/PC6	Loading/PC7
		Loadings for all investigated compounds, PC1-PC7						
**α-Thujene**	1	0.030741	0.533367	-0.353009	0.209095	-0.047703	0.511525	0.205284
**α-Pinene**	2	-0.716089	0.067542	-0.256083	-0.278049	-0.020502	0.353695	0.308816
**Camphene**	3	-0.418622	0.672674	-0.579813	-0.016626	-0.100803	0.092187	0.058049
**Sabinene**	4	-0.971969	-0.180686	-0.042770	-0.005839	-0.085202	0.069848	-0.011496
**β-Pinene**	5	-0.958696	-0.108659	-0.061235	0.029217	-0.079534	0.141601	0.041695
**β-Myrcene**	6	-0.975957	-0.170951	-0.017782	0.060166	-0.106909	0.013592	-0.007575
**3-Octanol**	7	0.065623	0.675279	-0.065993	-0.143509	-0.663086	0.064746	0.021727
**α-Phellandrene**	8	-0.976236	-0.049499	0.064999	0.006418	-0.055398	-0.157121	-0.033523
**α-Terpinene**	9	-0.917937	-0.066714	-0.008385	-0.303234	0.126169	0.021027	-0.139301
***para*-Cymene**	10	-0.980025	-0.080735	0.105628	-0.004776	0.015665	-0.069300	-0.088972
**Limonene (S-)**	11	0.009224	0.466445	-0.763158	0.075718	-0.232411	0.277275	0.020767
**1,8-Cineole**	12	-0.934298	-0.193698	0.077340	0.187955	-0.095798	-0.031471	-0.149248
***(Z)*-Ocymene**	13	0.415272	-0.708014	-0.387953	-0.109167	-0.284595	0.087360	-0.055810
***(E)*-Ocymene**	14	0.190051	-0.779617	-0.025507	-0.277106	-0.346585	-0.064466	-0.039047
**γ-Terpinene**	15	-0.958503	-0.059386	-0.002698	-0.166063	0.053222	-0.063091	-0.138226
**Terpinene-4-acetate**	16	-0.952447	-0.109294	0.135317	0.150459	-0.082928	-0.167321	-0.030265
**Linalool**	17	-0.707421	0.061724	-0.255784	-0.602311	0.114988	-0.042007	-0.147685
**3-Octyl acetate**	18	-0.898040	-0.092943	0.180193	0.249875	-0.078361	-0.174854	-0.055972
**Menthone**	19	0.546769	0.169262	0.647878	-0.246889	0.282971	-0.231147	0.212011
**Isomenthone**	20	0.492990	0.108175	0.453511	-0.162374	0.510022	-0.326126	0.309581
**Borneol**	21	0.098788	0.779189	-0.559564	-0.028277	-0.078457	-0.137930	0.062399
***Cis*-dehydro-α-terpineole**	22	-0.703782	-0.187247	0.231889	0.506585	-0.204155	-0.267982	0.161156
**Menthol**	23	0.368393	0.146826	0.652119	-0.377889	-0.435642	0.062195	-0.143936
***trans*-Isopulegone**	24	0.393883	0.186604	0.701832	-0.354912	-0.375299	0.059420	-0.104886
**Terpinene-4-ol**	25	-0.814867	-0.074890	-0.162271	-0.433570	0.123199	-0.046826	-0.230041
**Isomenthol**	26	-0.240007	0.000217	0.218616	0.464193	0.030098	-0.369481	-0.550745
**α-Terpineol**	27	-0.596478	-0.394071	0.044071	-0.030763	-0.274839	0.405339	0.381138
***cis*-Dihydrocarvone**	28	-0.055332	0.727988	-0.627070	0.022394	-0.158962	0.160451	0.035438
***trans*-Dihydrocarvone**	29	-0.044463	0.727524	-0.617217	0.040188	-0.160677	0.174957	0.045276
**Octanol acetate**	30	-0.529822	-0.108717	-0.099856	-0.674186	0.320441	0.213566	-0.212767
**1,6-Dihydrocarveol**	31	0.001745	0.608520	-0.585117	-0.043117	-0.234089	-0.222016	-0.101400
***trans*-Carveol**	32	-0.062443	0.397312	-0.476681	-0.179788	-0.201341	-0.474771	-0.206777
**carvenone**	33	0.278697	-0.704081	-0.435851	0.070566	-0.161688	0.026666	-0.058519
***cis*-Dihydrocarveol**	34	-0.062443	0.397312	-0.476681	-0.179788	-0.201341	-0.474771	-0.206777
**Citronellol**	35	-0.839215	-0.142851	0.158094	-0.003134	-0.105941	0.072413	0.364002
***cis*-3-Hexenyl isovalerate**	36	-0.662721	-0.141733	-0.015868	-0.473771	-0.070090	-0.211390	0.276025
**Pulegone**	37	0.312915	0.149585	0.637364	-0.370785	-0.489134	0.073177	-0.135666
**Carvone**	38	-0.003790	0.725479	-0.651111	0.018676	-0.219418	-0.032017	-0.024947
**Piperitone**	39	-0.036389	0.445921	-0.082711	-0.328783	0.548717	-0.069344	0.117806
***Cis*-Piperitone epoxide**	40	0.307598	-0.714431	-0.431420	0.192157	-0.067025	0.260809	-0.185589
**Citronellyl formate**	41	0.132055	-0.478138	-0.298974	-0.091986	-0.313703	-0.201793	0.298552
**Geranial; Citral A**	42	-0.469851	-0.060106	0.153408	0.183761	-0.203221	-0.021772	0.387536
**Neomenthyl acetate**	43	0.677304	-0.184582	0.524068	-0.352886	-0.170871	-0.132711	0.055098
**Dihydroedulan I.**	44	-0.165800	-0.290063	-0.619347	-0.185885	0.132529	-0.163003	-0.287610
**Menthyl acetate**	45	0.339469	0.147829	0.632383	-0.358521	-0.467624	0.090651	-0.177253
**Thymol**	46	-0.972715	-0.094191	0.034730	-0.068887	0.036307	-0.064315	-0.144991
**Carvacrole**	47	-0.757757	-0.101103	0.243685	0.506439	-0.189228	-0.235218	0.016137
**Dihydrocarvyl acetate**	48	0.010393	0.605485	-0.569312	-0.022257	-0.226318	-0.176006	-0.081756
**Citronellyl acetate**	49	-0.822902	-0.227076	0.104364	-0.025268	-0.132567	0.051082	0.376274
**Thymyl acetate**	50	-0.582231	-0.057917	0.019708	-0.694275	0.105736	0.163739	-0.137576
**Eugenol**	51	-0.713403	-0.145948	-0.011694	-0.468417	0.207584	0.190503	-0.045937
**Carvacryl acetate**	52	-0.738520	-0.082496	0.265629	0.523985	-0.159154	-0.252896	-0.047948
**β-Burbonene**	53	0.512182	-0.292767	-0.303753	-0.213675	0.332310	-0.241415	0.203289
**β-Elemene**	54	0.091024	-0.488994	-0.391137	-0.362261	-0.269583	-0.408533	0.271706
***cis*-Jasmone**	55	-0.619027	-0.205851	-0.119675	-0.712761	-0.014409	-0.002098	0.031128
**β-Caryophyllene**	56	-0.061425	-0.762830	-0.432633	0.257049	-0.018045	0.262003	-0.244980
**α-Humulene**	57	0.199671	-0.833029	-0.234907	0.335938	-0.101223	0.155498	-0.118234
**β-Farnesene**	58	0.290911	-0.316739	0.010492	0.085097	0.133048	0.371678	-0.456433
**Germacrene D**	59	0.412524	-0.605841	-0.595122	-0.113577	0.254230	0.052718	-0.011193
**1-Acetoxy-*p*-menth-3-one**	60	0.195772	-0.617406	-0.390895	-0.037561	-0.321301	-0.140194	0.240571
**Bicyclogermacrene**	61	0.601047	-0.186651	-0.596685	-0.169854	0.240706	-0.185919	0.151348
**β-Cadinene**	62	0.157583	-0.631405	-0.575627	-0.279728	-0.125803	-0.243509	-0.003280
**Spathulenol**	63	0.535147	-0.603199	-0.469936	-0.029409	-0.194269	-0.055398	-0.008577
**Caryophyllene oxide**	64	0.347559	-0.628030	-0.199679	-0.217522	-0.382759	-0.083543	0.012121
**Viridiflorol**	65	0.348019	0.153441	0.652079	-0.368083	-0.463548	0.087428	-0.139492

SUPPLEMENT 2

ORIGIN AND EXPERIMENTAL CONDITIONS OF THE FIVE *M. longifolia* ACCESSIONS

Table S3 Original locations of the five investigated ML accessions in Northern Hungary.

Accession	*Original habitat*			
	**Latitude**	**Longitude**	**Altitude**	**Characterization**
**KBÁ**	N 47.952612	E 19.815339	221	Wet glade between streambank and forests, near suburbia of *Bátonyterenye* town
**HV1**	N 48.029817	E 20.552683	239	Open meadows, 'puszta' at *Tebepuszta*; N.P. area
**HV2**	N 47.996033	E 20.513726	233	Forest glades near the rapids of *Hór* stream; N.P. area
**EGR3**	N 47.890970	E 20.390122	173	Streambank, urban ruderal area (edifices of a bridge) in suburbia of *Eger* city
**SZD**	N 48.088253	E 20.178323	398	Roadside between stream & cultivated area, in the close district of *Szentdomonkos*

N.P.=National Park. Measuring unit of latitude & longitude are degrees and of the latitude are metres *a.s.l.*

Table S4. Date of sampling of the horsemint accessions in their phenophase of full blooming.

**Accession**	**Habitat EGR**		**Habitat SOR**	
	**Year: 2019**	**Year: 2020**	**Year: 2019**	**Year: 2020**
**KBÁ**	05 Jul	08 Jul	02 Jul	25 Jun
**HV1**	30 Jun	08 Jul	02 Jul	25 Jun
**HV2**	30 Jun	10 Jul	02 Jul	25 Jun
**EGR3**	22 Jul	15 Jul	02 Jul	02 Jul
**SZD**	05 Jul	10 Jul	02 Jul	25 Jun

Table S5. Weather characteristics of the two habitats in the period of the blooming (20 Jun-25 Jul in each year)

Parameter	*Habitat EGR*		*Habitat SOR*	
	**Year 2019**	**Year 2020**	**Year 2019**	**Year 2020**
Mean temperature °C	21.6	20.5	21.5	20.8
Max. temperature °C	33.6	31.4	35.5	35.5
Min. temperature °C	9.6	9.1	5.6	6.3
Sum of mean temperatures °C	779.1	734.2	773.1	748.0
Precipitation mm	15.2	138.5	69.8	78.4

Table S6. Properties of soil at the two experimental habitats

**Parameter**	***Habitat EGR***	***Habitat SOR***
**Soil type**	Heavy loam	Sandy loam
**KA**, ml water/100 g	44	25
**pH**(H_2O)	7.97	7.82
**Humus** m/m%	2.84	2.08
NO_2^-+NO_3^-—**N** mg/kg	12.64	6.93
P_2O_5 mg/kg	678.1	412.9
K_2O mg/kg	439.5	412.9
Na^+ mg/kg	21.12	32.17
Cu^{2+} mg/kg	3.12	2.31
Mg^{2+} mg/kg	211.9	131.8
Mn^{2+} mg/kg	132.0	25.64
Zn^{2+} mg/kg	5.45	4.09
SO_4^{2-} mg/kg	39.5	55.7
$CaCO_3$ m/m%	0.34	0.32

KA= water absorption, ml water to 100 g soil till the maximal plasticity (*Arany* index of soil type and cohesion)
